# Methicillin-Resistant Coagulase Negative Staphylococci and Their Antibiotic Susceptibility Pattern from Healthy Dogs and Their Owners from Kathmandu Valley

**DOI:** 10.3390/tropicalmed6040194

**Published:** 2021-11-02

**Authors:** Muna Khanal, Prabhu Raj Joshi, Saroj Paudel, Mahesh Acharya, Komal Raj Rijal, Prakash Ghimire, Megha Raj Banjara

**Affiliations:** 1 Central Department of Microbiology, Tribhuvan University, Kirtipur, Kathmandu 44618, Nepal; khanalmuna22@gmail.com (M.K.); komal.rijal@cdmi.tu.edu.np (K.R.R.); prakash.ghimire@cdmi.tu.edu.np (P.G.); 2 Nepalese Farming Institute, Maitidevi, Kathmandu 44605, Nepal; cmilanjoshi@gmail.com (P.R.J.); pulu.saroj@gmail.com (S.P.); maheshacharya045@gmail.com (M.A.)

**Keywords:** MRCoNS, prevalence, human, dogs, SCC*mec*

## Abstract

This cross-sectional study was designed to identify information on the frequency, antimicrobial resistance and species diversity of methicillin-resistant coagulase negative staphylococci (MRCoNS) among pet dogs and humans within households. Fifty five nasal swabs each from dogs and their owners were collected. MRCoNS were identified based on gram staining, culture on mannitol salt agar, biochemical tests, and *mec*A gene amplification. The antibiotic susceptibility of the isolates was assessed by a disc diffusion test. Uniplex and multiplex polymerase chain reaction (PCR) were employed for the species identification of MRCoNS and SCC*mec* typing, respectively. Species were further confirmed by MALDI-TOF-MS. The prevalence of MRCoNS was 29% in dog owners and 23.6% in dogs. Four different species of MRCoNS, *Staphylococci saprophyticus* (48.3%), *S. haemolyticus* (24.1%), *S. warneri* (17.2%), and *S. epidermidis* (10.3%), were detected. Two isolates each from dog owners and dogs showed a constitutive resistance to macrolide-lincosamide-streptogramin B (cMLSB) resistance, eight isolates each from dogs and their owners showed a macrolide-streptogramin B (MSB) resistance, and only two isolates from dog owners revealed an inducible resistance to macrolide-lincosamide-streptogramin B (iMLSB) resistance. SCC*mec* types were SCC*mec* type IV (55.2%), SCC*mec* type V (24.1%), SCC*mec* III (10.3%), SCC*mec* II (3.4%); two isolates were non-typable. MRCoNS are prevalent and genetically diverse in companion animals and humans. Different species of MRCoNS were found in dogs and their owners.

## 1. Introduction

*Staphylococcus epidermidis*, a common coagulase negative Staphylococci (CoNS), has been implicated in human infections, and a majority of the strains circulating in hospitals has been estimated to be methicillin-resistant and additionally resistant to several classes of antibiotics [1]. Staphylococci acquire methicillin resistance by the acquisition of staphylococcal chromosomal cassette *mec* (SCC*mec*), the mobile genetic element carrying *mecA*, which encodes altered penicillin binding protein (PBP2a) that mediates resistance to virtually all beta-lactam antibiotics [2]. Besides *S. epidermidis*, other methicillin-resistant CoNS (MRCoNS), including *S. capitis*, *S. sciuri*, *S. hominis*, *S. haemolyticus*, *S. saprophyticus* and *S. warneri*, have also been reported from human clinical infections [3,4].

The use of many different classes of antibiotics in veterinary medicine for the treatment of infections has probably selected for an antibiotic-resistant commensal flora, including the colonization of healthy dogs and horses with MRCoNS [5,6]. Regardless of the pathogenic potential, MRCoNS have been identified as an important source of antibiotic resistance determinants; for instance, some strains of *S. xylosus* have been regarded as a potential source of the *mec*C gene encoded in methicillin-resistant *S. aureus* (MRSA) [7].

Antimicrobial resistance (AMR) in Nepal has increased rapidly since the past few decades [8]. In Nepal, companion animals such as dogs, which may be the source of antimicrobial-resistant bacteria or the corresponding resistance genes, live in close contact with humans. Additionally, stray dogs, which are more numerous in major towns of Nepal, including districts of Kathmandu, Bhaktapur, and Lalitpur, are also indirectly in contact with humans; close contact allows the transmission of bacterial agents including staphylococci. It is not clear yet to what extent Staphylococci may be shared between dogs and humans, which makes it important to investigate the potential colonization of human body sites with Staphylococci as well as to study the possible transfer of antibiotic resistance genes between dog and human Staphylococci. To test the association of these exchanges, we characterized MRCoNS from dogs and dog owners, and their antimicrobial resistance genes in this study.

## 2. Materials and Methods

### 2.1. Ethics Statement

This study received ethical approval from the Nepal Health Research Council (Regd. 457/2019 MT). Before the collection of nasal swab samples from humans and dogs, a written informed consent form was completed from humans. 

### 2.2. Research Design 

This was a cross-sectional quantitative study, and primary data were collected from February to July 2019. Samples were collected from pet dogs and their owners in the community and processed in the laboratory. 

### 2.3. Study Sites

The study was carried out in different communities of the Kathmandu, Bhaktapur and Lalitpur districts: these districts are highly populated, and human-dog contact is frequent. 

### 2.4. Sample Size and Sampling Technique

A total of 55 pairs (n = 110) of nasal swabs were collected from dogs and their owners, including 20 pairs each from the Kathmandu and Bhaktapur districts, and 15 pairs from the Lalitpur district. The nonduplicate random samples were collected from each population unit. 

### 2.5. Specimen Collection, Transport, and Identification of MRCoNS

Nasal swabs collected from dogs and their owners were kept in vials with M-Staph broth (HiMedia, India) enriched with a final concentration of 75 mg/L of polymyxin B, 0.01% potassium tellurite and 12.5 mg/L nystatin (Sigma-Aldrich, Massachusetts, MA, USA), screw-capped, clearly labeled and carried to the laboratory. Specimens were incubated inside a candle jar at 35 °C for 48 h. The broth was streaked in Mannitol salt agar plates and incubated at 35 °C for 24 h. Both mannitol fermenter and nonfermenter, at 2–3 colonies per sample, were further sub-cultured on nutrient agar. The bacteria were identified based on colony morphology, mannitol fermentation, Gram staining and conventional biochemical tests including catalase, oxidase, coagulase tests and DNAase activity. Isolates were further tested for methicillin resistance by growth on Mueller–Hinton Agar (MHA) containing 4 mcg/mL oxacillin, a cefoxitin (30 mcg) disc diffusion test and the PCR amplification of the *mec*A gene [9,10].

### 2.6. Antibiotic Susceptibility Test 

MRCoNS isolates were subjected to an in-vitro antimicrobial susceptibility test by the disc diffusion method with the following antibiotics: gentamicin (10 µg), erythromycin (15 µg), ciprofloxacin (5 µg), tetracycline (30 µg), clindamycin (2 µg), co-trimoxazole (25 mcg), linezolid (30 µg) and chloramphenicol (50 µg) (HiMedia, India). The results were interpreted following the Clinical and Laboratory Standard Institute guidelines M100 (CLSI 2019). The minimum inhibitory concentration (MIC) of oxacillin (0.125 to 64 mg/L) to the bacterial isolates was determined by agar dilution method. Briefly, colonies isolated from the overnight growth of each organism were selected to prepare direct suspensions in tryptic soy broth. The suspensions were adjusted to a 0.5 McFarland standard. Control Mueller–Hinton agar and oxacillin Mueller–Hinton agar plates were inoculated with the final suspensions of bacteria. Plates were incubated at 35 °C for 24 h. The MIC was determined as the lowest concentration of oxacillin that completely inhibited growth. Erythromycin-resistant and clindamycin-susceptible isolates were also tested for inducible clindamycin resistance by D zone test [9]. 

### 2.7. Species Identification of MRCoNS and SCCmec Typing

The species identification of MRCoNS was carried out by uniplex PCR using primer sequences and optimization conditions, as described previously (Table 1). A multiplex PCR assay was employed to characterize the isolates by SCC*mec* typing [10]. The primer sets are listed in Table 2. The amplified PCR products were analyzed through electrophoresis on a 1.5% agarose gel. 

The species of MRCoNS were further confirmed by MALDI-TOF-MS, as described by Kitti et al [11]. Briefly, several colonies from MHA were harvested and collected in 100 μL of sterile water. One microliter of this aliquot was deposited on two replicated target plates (Bruker Daltonics, Germany), being allowed to dry at room temperature. Then, one microliter of absolute ethanol (Merck, Darmstadt, Germany) was added to each well. Following drying at room temperature, 1 μL of α-cyano-4-hydroxycinnamic acid (Bruker Daltonics, Germany) dissolved in a solution of 50% acetonitrile, 2.5% trifluoroacetic acid, and 47.5% water (Sigma-Aldrich, Fluka, MO, USA) was added as a matrix. Both MALDI-TOF-MS Spectrometer Autoflex speed (Bruker Daltonics, Germany) and FlexControl software (version 3.4.135, Bruker Daltonics, Germany) were processed for the detection of protein and identification of the difference between species. The scores were used for the genus- and species-level identification: a score of 2000–3000 specified a species-level identification, while a score ranging from 1700–1999 indicated a genus-level identification, and those strains with a score below 1700 had an unreliable identification. The strains which could not be identified by MALDI-TOF MS were not processed further. 

### 2.8. Data Analysis

Data were entered into SPSS version 21.0. A chi-square test was used to determine the association of human isolates and dog isolates. A p-value less than 0.05 was considered significant.

## 3. Results

### 3.1. Nasal Carriage Rate of MRCoNS

From the 55 human nasal samples, 16 (29%) were positive for MRCoNS; nine and seven isolates were from Kathmandu and Bhaktapur, respectively. Similarly, out of 55 nasal samples from dogs, 13 (23.6%) MRCoNS were isolated, and the distribution was: Kathmandu (n = 2), Bhaktapur (n = 5) and Lalitpur (n = 6).

### 3.2. Antibiotic Susceptibility of MRCoNS 

The antibiotic susceptibility of MRCoNS isolated from dog owners (n = 16) and dogs (n=13) revealed an almost similar resistance pattern to erythromycin (75% vs. 77%), co-trimoxazole (31.2% vs. 38.5%), and ciprofloxacin (18.8% vs. 23.1%). MRCoNS isolates from dog owners were also resistant to clindamycin (18.8%), gentamicin (12.5%) and chloramphenicol (6.3%). None of the MRCoNS isolates from dog owners showed resistance to tetracycline and linezolid. Likewise, MRCoNS isolated from dogs were resistant to clindamycin (15.4%) and tetracycine (15.4%), and were fully susceptible to linezolid (Table 3). 

The MIC of oxacillin to MRCoNS were determined and found to be 4 µg/mL to 32 µg/mL; isolates from dog owners had higher MIC of oxacillin than isolates from dogs (Table 4). 

Isolates were also tested for whether they had constitutive and/or inducible clindamycin resistance. Four MRCoNS isolates, two each from dog owners and dogs, showed a constitutive resistance to macrolide-lincosamide-streptogramin B (cMLS_B_) resistance. Likewise, 16 isolates, eight each from dogs and their owners, revealed MS_B_ resistance_._ Only two isolates from dog owners showed an inducible resistance to macrolide-lincosamide-streptogramin B (iMLS_B_) resistance (Table 5).

### 3.3. mecA Gene in MRCoNS, Species and SCCmec Types of MRCoNS from Dog Owners and Dogs

The *mec*A gene was detected among all the 29 isolates of MRCoNS that were resistant to cefoxitin (30 µg) and oxacillin (MIC > 4 µg/mL). The species distribution of 16 MRCoNS from dog owners were *S*. *saprophyticus* (n = 9; 56.3%), *S*. *haemolyticus* (n = 3; 18.8%), *S*. *epidermidis* (n = 2; 12.5%) and *S*. *warneri* (n = 2; 12.5%). Similarly, four different species of MRCoNS were detected from dogs, including *S*. *saprophyticus* (n = 5; 38.5%), *S*. *haemolyticus* (n = 4; 30.8%), *S*. *warneri* (n = 3; 23.1%), and *S*. *epidermidis* (n = 1; 7.7%) (Table 6). 

The MRCoNS isolates were further characterized by SCC*mec* typing. Most strains were SCC*mec* type IV (55.2% n = 16), seven (43.7%) from dog owners and nine (69.2%) from dogs. Similarly, SCC*mec* type V were 24.1% (n = 7), five (31.3%) from dog owners and two (15.4%) from dogs. In total, SCC*mec* III were 10.3% (n = 3), SCC*mec* II were 3.4% (n = 1), and two isolates were non-typable (Table 6). 

### 3.4. Coexistence of MRCoNS in Dogs and Their Owners

The same species of MRCoNS was not found to coexist in pairs; however, one sample from Bhaktapur had MRCoNS from both the pets and their owners, but from different species: *S. warneri* was found in dogs and *S. saprophyticus* in humans. 

## 4. Discussion

This study provides information on the frequency, antimicrobial resistance and species diversity of MRCoNS within households, as well as on MRCoNS pet–human interaction. From this study, we found that the prevalence of MRCoNS was slightly higher in dog owners than in dogs. *S*. *saprophyticus* was found to be the dominant species in both dogs and dogs’ owners. MRCoNS isolates, both from dogs and their owners, showed resistance to ciprofloxacin, co-trimoxazole, chloramphenicol, clindamycin, erythromycin and gentamicin in varying proportions, and all the MRCoNS were susceptible to linezolid among the tested antibiotics. The molecular characterization of the isolates revealed the presence of the *mec*A gene among all MRCoNS, and most isolates carried the SCC*mec* IV element. The same strains of MRCoNS were not found to be shared among dogs and their owners in this study. 

The carriage rate of MRCoNS detected among healthy owners (29%) was higher than those reported in previous studies among healthy individuals (7–28%) in non-healthcare settings [16,17,18,19,20,21]. Jamaluddin et al. detected a higher rate of MRCoNS carriage (30%) in Japanese children in day-care centers and kindergartens [22], and one study also reported a 47–51% carriage of MRCoNS among a remote population in French Guiana [23]. The data from all of these studies show that the types of cohort markedly determine the nasal carriage of MRCoNS. Very few data are available on the nasal MRCoNS carriage rate and associated risk factors. In one study, the MRCoNS prevalence in healthy humans that were in daily contact with a companion animal was 54.2%, which suggested a direct pet–human contact as a risk factor of a higher MRCoNS carriage [24]. The carriage of MRCoNS among pets is also in the variable range: lower rates (1–15%) than those detected in our study (23.6%) have been reported among healthy dogs from nasal, rectal, oral, anal and belly sites [6,25,26,27,28,29], and a very high rate (42%) has been reported in nasal and perineal samples taken from healthy non-vet-visiting and non-antibiotic-treated Labrador retrievers [30], indicating that the observed prevalence may be affected by different sampling methodologies. 

In contrast with the former studies, which have detected *S. epidermidis* as a predominant staphylococcal species colonizing human body sites [1,16,30], we detected four species of staphylococci, *S. saprophyticus S. haemolyticus, S. epidermidis* and *S. warneri,* in decreasing proportions. The species distribution of human MRCoNS reported in our study seems diverse, even in similar geographic locations. Different species of MRCoNS have been detected among the dogs, including *S. sciuri*, *S. warneri, S. lentus*, *S. vitulinus*, or *S. fleurettii* and *S*. *epidermidis* [1,16,26,27,29,30]. In agreement with these findings, our study also detected diverse species of MRCoNS in dogs. The exact cause of the dominance of *S. saprophyticus* over other species of MRCoNS in both humans and dogs, however, needs further investigation.

SCC*mec* types having a high diversity were detected, most being SCC*mec* IV (55.2%), which was in agreement with former studies that also detected SCC*mec* IV as a prevalent cassette among companion animals and humans [1,17,20,23,31,32,33,34,35,36,37]. This study observed two *mec*A positive *S. warneri* non-typable for the SCC*mec* element, and this is common in MRCoNS strains because of the high variability of the SCC*mec* element and lack of primers that target all *ccr* and *mec* genes. The whole genome sequencing is definitely needed for an in-depth analysis [16]. The lack of typeability, highly diverse SCC*mec*, and presence of novel *mec* and *ccr* genes’ complex combination reveal an increasing complexity in the SCC*mec* typing of MRCoNS from humans and companion animals. Such mobile segments may be an important source of resistance determinants to other staphylococci that reside in the same niche. 

Erythromycin, an important antimicrobial used for staphylococcal infections in both human and animals, was the drug for which most MRCoNS from humans and dogs exhibited resistance. The macrolides-lincosamides-streptogramins (MLS) resistance in staphylococci is not surprising [25,28,32,37,38]. The resistance to both erythromycin and clindamycin is common in *S. aureus*, but most MRCoNS strains exhibit resistance to either erythromycin or clindamycin, as shown in our study and in another previous report [39]. This shows the differential ability of *S. aureus* and MRCoNS to acquire resistance genes. We also detected MRCoNS isolates resistant to co-trimoxazole and ciprofloxacin in almost equal proportion, and to the other antibiotics including gentamicin and chloramphenicol in varying proportions. These antibiotics are used in both human as well as veterinary medicine in Nepal, and resistance to these drugs may be attributed to their frequent use. We detected only two isolates of MRCoNS from dogs that were resistant to tetracycline, and staphylococci resistance to this drug is low. The linezolid-resistant strain of methicillin-resistant *S. aureus* (MRSA) has been reported in one study carried out by Roberts et al in Nepalese swines [40]. This study did not report linezolid-resistant MRCoNS, which shows that strains of staphylococci resistant to this drug are uncommon in Nepal.

Studies targeting the transmission dynamics of MRCoNS between dogs and their owners have found that the same strain of CoNS is shared among these two hosts, even though the occurrence is very low [16,41]. However, this study did not find any sharing of the same strain to occur between dogs and their owners. This may be attributed to the low sample size and limited sample types; a large-scale study with multiple sample types should be done in order to warrant the conclusion. We could not perform pulse-field gel electrophoresis (PFGE), multi-locus sequence typing (MLST), spa typing, and whole genome sequencing (WGS) of the strains, which could provide an epidemiological pattern of the transmission of MRCoNS. All the dogs that were included in this study were kept in the house; however, data regarding their age, breeds, and captivity were not available for comparison.

## Figures and Tables

**Table 1:** Primers used in the species identification of MRCoNS.

Primers.	Oligonucleotide Sequence (5’-3’ )	Ta (°C)	Product Size (bp)	References
nuc (*S. warneri*)	F-CGTTTGTAGCAAAACAGGGC R-GCAACGAGTAACCTTGCCAC	60	999	[12]
rdr (*S. epidermidis*)	F-AAGAGCGTGGAGAAAAGTATCAAG R-TCGATACCATCAAAAAGTTGG	62	130	[13]
groESL-F (*S. haemolyticus*)	F-GGTCGCTTAGTCGGAACAAT R-CACGAGCAATCTCATCACCT	58	271	[14]
sodA (*S. saprophyticus*)	F-TCAAAAAGTTTTCTAAAAAATTTAC R-ACGGGCGTCCACAAAATCAATAGGA	55	580	[15]

**Table 2:** Primers used in the multiplex SCC*mec* PCR.

Primer Sequence (5’-3’)	Target	Length (bp)	SCC*mec* Type
F-ATTGCCTTGATAATAGCCYTCT R-TAAAGGCATCAATGCACAAACACT	ccrA2-B	937	II, IV
F-CGTCTATTACAAGATGTTA AGGATAAT R-CCTTTATAGACTGGATTATTCAAAATAT	CcrC	518	III, V
F-GCCACTCATAACATATGGAA R-CATCCGAGTGAAACCCAAA	IS1272	415	I, IV
F-TATACCAAACCCGACAACTAC R-CGGCTACAGTGATAACATCC	mecA–IS431	359	V

**Table 3:** Antibiotic susceptibility pattern of MRCoNS in dog owners and dogs.

Antibiotics.	Susceptibility Patterns (n = 16)			Susceptibility Patterns (n = 13)		
	(Dog owners)			(Dogs)		
	S (%)	I (%)	R (%)	S (%)	I (%)	R (%)
Erythromycin	4 (25)		12 (75)	3 (23)		10 (77)
Clindamycin	13 (81.2)		3 (18.8)	11 (84.6)		2 (15.4)
Co-trimoxazole	10 (62.5)	1 (6.3)	5 (31.2)	8 (61.5)		5 (38.5)
Ciprofloxacin	13 (81.2)		3 (18.8)	10 (76.9)		3 (23.1)
Gentamicin	14 (87.5)		2 (12.5)	11 (84.6)	1 (7.7)	1 (7.7)
Chloramphenicol	15 (93.7)		1(6.3)	12 (92.3)		1 (7.7)
Tetracycline	16 (100)		0	11 (84.6)		2 (15.4)
Linezolid	16 (100)		0	13 (100)		0

**Table 4:** MIC of oxacillin to MRCoNS isolated from dogs and their owners.

MRCoNS (Dog)	MICs (µg/mL)	MRCoNS (Human)	MICs (µg/mL)
D1	4	M1	16
D2	4	M2	16
D3	16	M3	32
D4	16	M4	32
D5	16	M5	32
D6	4	M6	8
D7	8	M7	16
D8	16	M8	8
D9	16	M9	8
D10	16	M10	4
D11	16	M11	32
D12	4	M12	32
D13	16	M13	32
		M14	16
		M15	4
		M16	32

**Table 5:** Inducible and constitutive MLS_B_ pattern in dogs and dog owners.

Total	iMLS_B_ Pattern	cMLS_B_ Pattern	MS_B_ Pattern	Susceptible
Number				Pattern
Dog owner (16)	2 (12.5%)	2 (12.5%)	8 (50%)	4 (25%)
Dog (13)	0	2 (15.4%)	8 (61.5%)	3 (23.1%)

**Table 6:** Species and SCC*mec* types of MRCoNS from dog owners and dogs.

SCC*mec* Type	Isolates from Dog Owners					Isolates from Dogs				
	Total n (%)	*S. saprophyticus*	*S. haemolyticus*	*S. warneri*	*S. epidermidis*	Total n (%)	*S. saprophyticus*	*S. haemolyticus*	*S. warneri*	*S. epidermidis*
*I*	0	-	-	-	-	0	-	-	-	-
*II*	1 (6.3)	1	-	-		0	-	-	-	-
*III*	2 (12.6)	1	-	-	1	1 (7.7)	1	-	-	-
*IV*	7 (43.7)	3	2	1	1	9 (69.2)	3	3	2	1
*V*	5 (31.3)	4	1	-	-	2 (15.4)	1	1	-	-
*Non-typable*	1 (6.3)	-	-	1	-	1 (7.7)	-	-	1	-
Total	16	9 (56.3%)	3 (18.8%)	2 (12.5%)	2 (12.5%)	13	5 (38.5%)	4 (30.8%)	3 (23.1%)	1 (7.7%)

## Data Availability

All relevant data has been presented in the manuscript.
